# Astonishing improvement of destructive mandibular osteomyelitis by NSAIDs: A rare lesion site in a patient with SAPHO syndrome

**DOI:** 10.1002/ccr3.2328

**Published:** 2019-07-27

**Authors:** Masaki Tago, Naoko E. Katsuki, So Motomura, Yoshinori Tokushima, Shu‐ichi Yamashita

**Affiliations:** ^1^ Department of General Medicine Saga University Hospital Saga Japan

**Keywords:** SAPHO syndrome, sterile mandibular osteomyelitis

## Abstract

When patients develop destructive osteomyelitis, clinicians must always consider the possibility of SAPHO syndrome because even extremely destructive osteomyelitis can be cured by NSAIDs.

## 1 CASE

**Figure 1:**
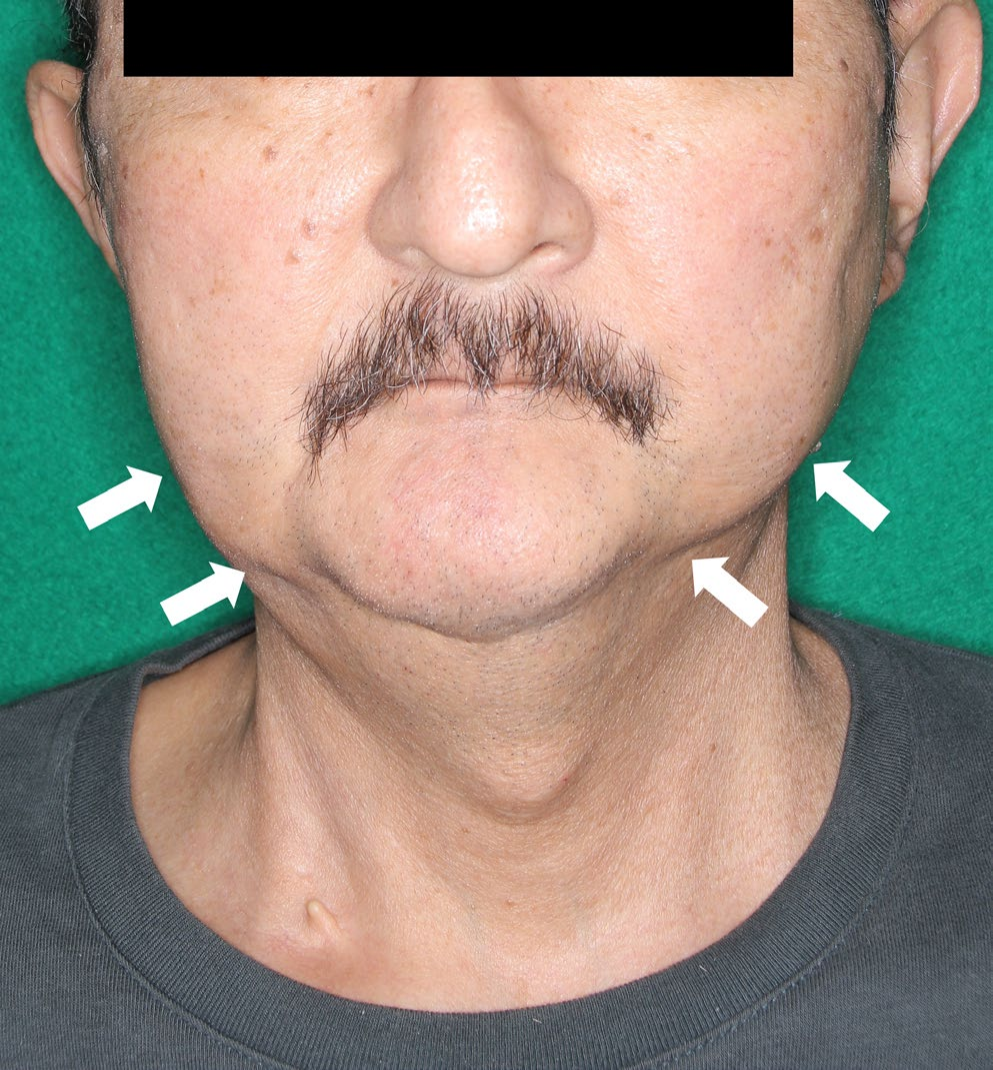
Findings of bilateral mandibles. Swelling and deformation of the bilateral mandibles were present (arrows)

**Figure 2:**
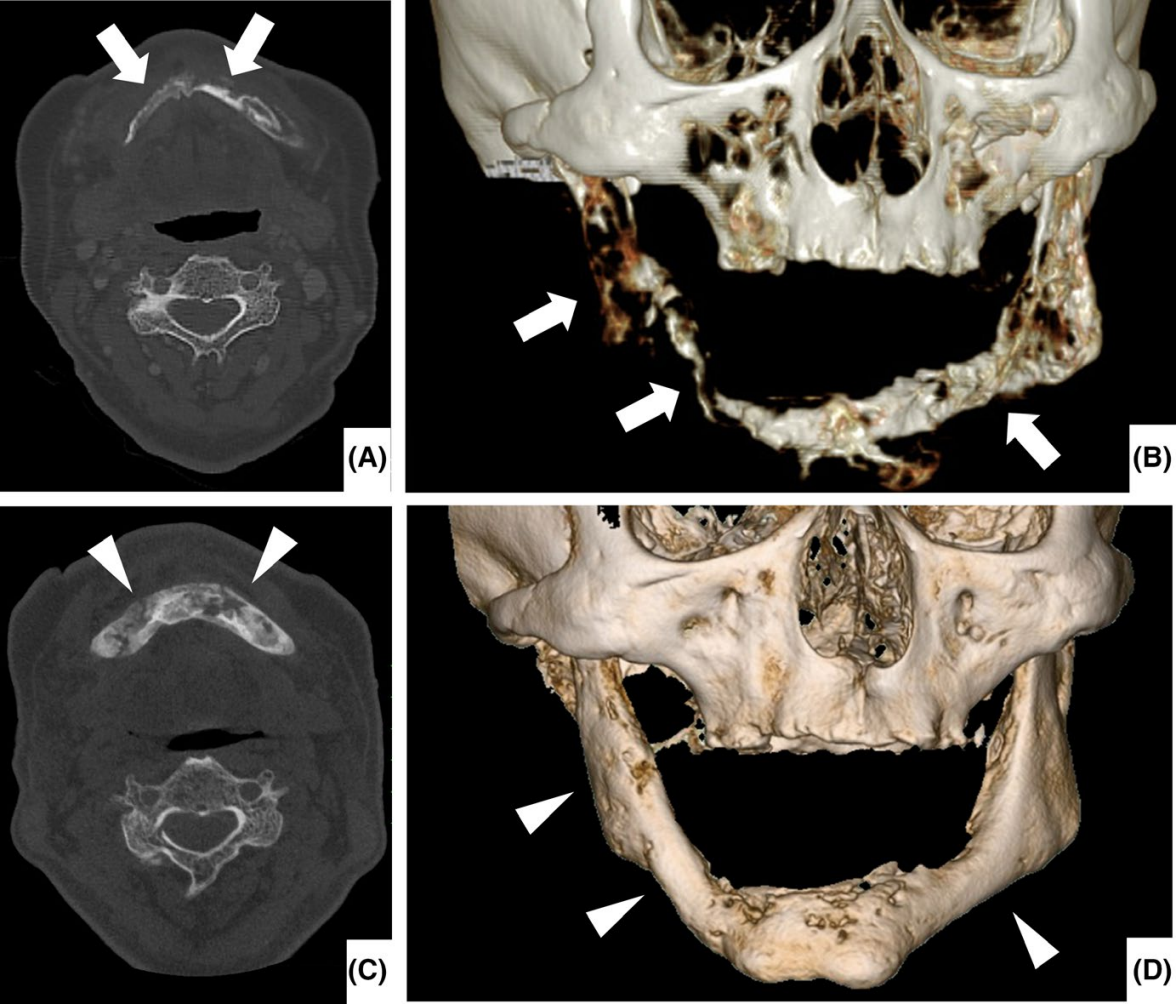
Facial computed tomography performed (A, B) on the first visit to our department and (C, D) 4 mo later. A, Cross‐sectional image shows sclerosing changes around marked osteolytic changes on both sides of the mandibular bones (arrows). B, Reconstructed image more clearly shows osteolytic changes on both sides of the mandibular bones (arrows). C, Cross‐sectional image shows marked improvement of sclerosing and osteolytic changes (arrowheads). D, Reconstructed image shows marked regeneration of the mandibular bones (arrowheads)

A 61‐year‐old man with a long history of repetitive inflammatory acne had a 24‐month history of pain, burning sensation, and swelling of the bilateral mandibles (Figure 1). He was diagnosed with mandibular osteomyelitis because of sclerosing changes around marked osteolytic changes in his bilateral mandibles on computed tomography (CT) (Figure 2A, B). Bone scintigraphy performed 11 months before admission revealed low uptake in the right mandibular bone, lower thoracic vertebrae, and bilateral sternoclavicular joints. On admission, his right lower jaw showed swelling without tenderness of the clavicular bones. CT and magnetic resonance imaging of the axial bones revealed a new osteolytic lesion on the left clavicle. He was diagnosed with SAPHO syndrome based on the presence of idiopathic sterile osteomyelitis, chronic recurrent multifocal osteomyelitis, and a long history of repetitive inflammatory acne after exclusion of infection or malignancy. By a widely acknowledged standard regimen for the treatment of SAPHO syndrome with etodolac (400 mg/day) and minocycline (200 mg/day) for 4 months, his pain and inflammatory markers showed definite improvements with marked regeneration of mandibular bone on CT (Figure 2C, D).

SAPHO syndrome must be considered in patients with sterile osteomyelitis, even in unusual sites,^1^ and especially with a history of skin involvement or even slight abnormalities within axial bones in imaging tests.

## CONFLICT OF INTEREST

The authors state that they have no conflict of interest.

## AUTHOR CONTRIBUTION

MT and NEK: involved in literature search, study conception, and manuscript drafting.SM: involved in literature search and clinical care of the patient. YT: involved in manuscript drafting and clinical care of the patient. SY: involved in study conception and manuscript revision.
